# Sodium Pyruvate Supplementation Enhances Infectious Yield and Supports Host-Cell Stability of Rabies Virus CVS-11 in a High-Density Macrocarrier-Based Tide-Motion Culture System

**DOI:** 10.3390/v18060600

**Published:** 2026-05-26

**Authors:** Tolganay Imanbekova, Nurlan Akhmetsadykov, Bakdaulet Shanbayev, Zhanat Batanova, Ernur Nurolda, Yerkin Krykbayev, Anara Nurmukhambetova, Hsian-Yu Wang, Yu-Jing Zeng

**Affiliations:** 1 Kazakh National Agrarian Research University, 8 Abai Avenue, Almaty 050010, Kazakhstan; zhanat.batanova@gmail.com; 2 Research and Production Enterprise Antigen LLP, Azerbayev Str. 4, Almaty Region, Settlement Abay, Karasai District, Almaty 040905, Kazakhstanbagdi76.kz@gmail.com (B.S.); nurolda.ernur@gmail.com (E.N.); erkinkrykbaev8@gmail.com (Y.K.); 3 Asfendiyarov Kazakh National Medical University, Tole Bi Street 94, Almaty 050012, Kazakhstan; anara.baubekovna.05@gmail.com; 4 Institute of Animal Vaccine Technology, National Pingtung University of Scientific and Technology, Taiwan R.O.C. 1, Shuefu Road, Neipu, Pingtung 912301, Taiwan; hyw@mail.npust.edu.tw (H.-Y.W.); yjz@mail.npust.edu.tw (Y.-J.Z.)

**Keywords:** rabies virus, CVS-11, Vero cells, BSR cells, sodium pyruvate, serum-free culture, high-density culture, metabolic modulation

## Abstract

Efficient in vitro production of rabies virus is essential for vaccine development and quality control applications. High-density cultivation systems offer practical advantages for rabies virus production but also create culture conditions in which nutrient depletion, waste accumulation, and progressive deterioration of host-cell condition may limit infectious virus output. In this study, we evaluated the effects of sodium pyruvate supplementation on rabies virus CVS-11 production in Vero and BSR cells cultivated in a high-density macrocarrier-based tide-motion culture system under serum-containing and serum-free conditions, with complementary comparative observations in conventional monolayer cultures of BHK cells. Cultures were infected at a multiplicity of infection of 0.01, and infectious virus production was assessed over time, together with cell density, glucose consumption, and pH dynamics. Sodium pyruvate supplementation was associated with significantly higher infectious virus titers, delayed culture deterioration, prolonged maintenance of viable cell populations, and higher peak infectious titers in both Vero and BSR cultures. The highest infectious titers were observed under serum-free pyruvate-supplemented conditions, reaching 7.5 log_10_ FFU/mL in Vero cells and 7.2 log_10_ FFU/mL in BSR cells. Across the tested conditions, serum-free cultivation and pyruvate supplementation were both associated with significantly higher peak infectious titers. In contrast, exploratory correlation analysis based on condition-level summary values indicated an inverse association between minimum culture pH and peak infectious titer. Together, these findings show that sodium pyruvate supplementation can improve infectious rabies virus yield and prolong the productive phase in high-density macrocarrier-based cultures, supporting its use as a practical culture-modulation strategy for CVS-11 production in adherent cell systems.

## 1. Introduction

Rabies remains a fatal zoonotic disease of major global public health importance, causing tens of thousands of human deaths annually despite the availability of effective prophylactic vaccines [1,2]. Sustained rabies control depends on the large-scale production of safe and immunogenic inactivated vaccines. In this context, viral antigen yield is influenced not only by process design but also by the biological properties of the host cell, including its permissiveness to infection and metabolic state [3,4,5]. Accordingly, current rabies vaccine development and manufacturing are increasingly supported by advances in cell culture technologies and biologically informed cultivation strategies that promote stable virus replication under controlled, scalable, and regulatory-compliant conditions [6,7].

The challenge virus standard strain CVS-11 is a well-established rabies virus strain widely used in vaccine research and quality control because of its stable antigenic characteristics and reproducible replication in permissive cell substrates, making it a suitable model for investigating virus–host interactions under controlled conditions [8,9,10]. Continuous cell lines such as Vero and BSR cells are commonly used for rabies virus propagation because of their well-characterized biological properties, high susceptibility to infection, and broad regulatory acceptance [11,12]. However, conventional monolayer systems, including T-flasks and roller bottles, impose inherent biological and engineering limitations, including restricted growth surface area, limited attainable cell density, and progressive changes in the cellular microenvironment that may reduce viral replication efficiency, particularly under serum-free conditions [13,14].

Recent advances in cultivation technology have enabled high-density propagation of adherent cells in macrocarrier-based systems, creating cellular environments characterized by increased cell density and altered nutrient and waste gradients [15,16]. Tide-motion platforms, such as the CelCradle™ system, provide an expanded growth surface under low-shear conditions and support efficient nutrient exchange, thereby helping to maintain cell viability in dense cultures [15,17]. Beyond their technological utility, such systems also provide a useful experimental framework for studying virus replication in host-cell populations under density-dependent metabolic constraints. Although macrocarrier-based cultures have been successfully applied to the production of several viral vaccines and vectors [18], systematic investigation of how host-cell metabolic state influences rabies virus replication under high-density conditions remains limited [19,20,21,22].

The metabolic state of the host cell is a major biological factor influencing viral replication, as virus propagation depends on cellular energy metabolism and biosynthetic capacity [22]. In high-density adherent cultures, progressive nutrient depletion, accumulation of metabolic by-products, and altered microenvironmental conditions may reduce cellular permissiveness and shorten the productive phase of infection [23]. Sodium pyruvate, a common medium supplement and central intermediate of cellular carbon metabolism, has been reported to support oxidative metabolism and cell viability under metabolically demanding culture conditions [24]. However, despite its widespread use in cell culture media, its specific role in supporting rabies virus replication under serum-free, high-density conditions has not been clearly defined [25,26].

In this study, we investigated the production of rabies virus CVS-11 in Vero and BSR cells cultivated under high-density adherent conditions in a tide-motion macrocarrier-based system as a model of density-associated culture constraint. We evaluated whether sodium pyruvate supplementation could improve culture performance by prolonging the productive phase and enhancing infectious virus yield under serum-free and serum-containing conditions. In addition, comparative monolayer experiments including BHK cells were used to place post-infection cell-density responses in a broader cell-substrate context. We hypothesized that targeted metabolic support would improve culture robustness during the post-infection phase and thereby support sustained infectious virus production.

This study demonstrates that sodium pyruvate supplementation is associated with prolonged maintenance of viable host-cell populations and higher infectious CVS-11 yields in high-density macrocarrier-based cultures. At the same time, comparative monolayer observations across Vero, BSR, and BHK cells provide a broader context for these effects.

## 2. Materials and Methods

### 2.1. Cell Lines and Virus Strain

Continuous Vero and BSR cell lines were used as host substrates for rabies virus propagation. Cells were maintained at 37 °C in a humidified atmosphere containing 5% CO_2_ and were routinely monitored for morphology and contamination. The challenge virus standard strain CVS-11 was used throughout the study. Before the high-density cultivation experiments, the virus was adapted to replication in Vero and BSR cells by serial passage under defined culture conditions. A total of 8 sequential passages were performed to ensure stable growth characteristics and reproducible infection kinetics. Continuous Vero, BSR, and BHK cell lines were used in this study. Vero and BSR cells served as the principal host substrates for high-density macrocarrier-based cultivation and virus-production analyses. In contrast, BHK cells were included in complementary monolayer experiments to compare post-infection cell-density responses across cell substrates under conventional cultivation conditions.

### 2.2. Culture Media and Supplements

Cell cultivation was performed in Dulbecco’s Modified Eagle Medium (DMEM) without sodium pyruvate (Capricorn Scientific, Ebsdorfergrund, Germany). Medium concentrations are expressed as the amount of powdered DMEM formulation per liter, as DMEM represents a complex multi-component formulation rather than a single defined compound and cannot be accurately described in molar units. During the expansion phase, the medium was supplemented with 10% fetal bovine serum and antibiotics, including gentamicin sulfate (50 μg/mL) and a penicillin–streptomycin mixture (100 U/mL penicillin and 100 μg/mL streptomycin). For serum-free experiments, cultures were transitioned to serum-free DMEM immediately before virus inoculation. In selected experimental conditions, sodium pyruvate was added as a defined metabolic supplement from a sterile-filtered stock solution (100 mM; Capricorn Scientific, Germany) to a final concentration of 1 mM. All media components and supplements were of cell culture grade.

### 2.3. Tide-Motion Macrocarrier-Based System Cultivation System

High-density cultivation of adherent cells was performed in a tide-motion macrocarrier-based system (CelCradle™/BelloCell^®^ 500 A configuration) using BioNOC™ II macrocarriers (Cesco Bioengineering Co., Ltd., Taiwan) [27]. The working volume was 500 mL. Each unit contained 5.5 g of macrocarriers (approximately 850 units), corresponding to an estimated total growth surface area of ~13,200 cm^2^. Cultivation was conducted at 37 °C in a humidified atmosphere with 5% CO_2_. Tide-motion settings were adjusted to provide periodic wetting of the macrocarriers and stable nutrient and gas exchange throughout cultivation.

### 2.4. Cell Seeding, Attachment, and Expansion

Cells were initially expanded in conventional T-flasks, detached with Versene (0.02%) and trypsin (0.25%), collected by centrifugation (1800 rpm, 5 min), and resuspended in growth medium. Cell suspensions were counted and inoculated onto BioNOC™ II macrocarriers at a density of 2 × 10^5^ cells per macrocarrier (cells/carrier).

For the attachment phase, the cultivation bottle was positioned upside down, and the macrocarriers were covered with 30–40 mL of DMEM supplemented with 10% fetal bovine serum (Capricorn Scientific, Ebsdorfergrund, Germany). The bottle was gently agitated every 30–60 min to promote uniform cell attachment. After 1–3 h, attachment efficiency was determined by counting the non-attached cells remaining in suspension and calculating the percentage of attached cells as follows:

Attachment efficiency (%) = [1 − (non-attached cells/total seeded cells)] × 100

If attachment efficiency exceeded 85%, the medium volume was adjusted to 500 mL, and cultivation was switched to tide-motion operation using the following settings: rise speed, 1.5 mm/s; top hold, 30 s; down speed, 1.5 mm/s; bottom hold, 30 s.

### 2.5. Serum-Free Transition and Infection Strategy

For serum-free infection experiments, cultures were transferred to serum-free DMEM immediately before virus inoculation. The timing of infection was guided by culture status and metabolic indicators, including glucose consumption [10].

In the macrocarrier-based system containing 5.5 g of BioNOC™ II macrocarriers, inoculation was performed using a virus suspension adjusted to achieve the target multiplicity of infection (MOI). Cultures were infected with CVS-11 at a defined MOI of 0.01, calculated based on the total number of cells present at the time of infection. The inoculum volume and virus concentration were adjusted accordingly to ensure consistent infection conditions across experimental replicates.

Under these conditions, the inoculum corresponded to approximately 1.1 × 10^6^ infectious units of CVS-11 in 50 mL of medium. This standardized approach enabled reproducible infection kinetics across different culture conditions.

### 2.6. Virus Harvest and Sample Handling

Virus-containing supernatants were collected at predefined time points during the productive phase of infection. Supernatants were clarified by low-speed centrifugation to remove cell debris and macrocarrier fragments, aliquoted, and stored at −70 °C until further analysis. During medium-exchange regimens, approximately 90% of the culture supernatant was removed and replaced with fresh medium, as specified by the experimental condition [18]. Harvesting was continued until a pronounced decline in infectious titer and/or substantial loss of cell viability was observed. Samples for glucose measurement and virus titration were collected at regular intervals during cultivation and infection, corresponding to key stages of cell growth and virus production.

### 2.7. Conventional Monolayer Cultivation (T-Flask Controls)

Parallel control experiments were performed in conventional T-flask monolayer cultures of Vero and BSR cells. Cell expansion, transition to serum-free conditions, infection with CVS-11, and virus harvest were conducted according to the same general infection framework used for the macrocarrier-based experiments. These controls enabled direct comparison between high-density macrocarrier-based cultivation and conventional monolayer culture systems.

### 2.8. Infectious Titer Determination

Infectious virus titers were quantified using cell-based infectivity assays and expressed as log_10_ TCID_50_/mL and/or log_10_ FFU/mL. Serial 10-fold dilutions of virus-containing samples were prepared and inoculated onto susceptible Vero cell monolayers. For TCID_50_ assays, cytopathic effects were evaluated after 96 h of incubation, and titers were calculated using the Reed–Muench method. For FFU assays, infected cells were detected by immunostaining with FITC-labeled anti-rabies virus antibodies (Capricorn Scientific, Germany), and infectious foci were counted to calculate FFU/mL [10]. To ensure comparability across experiments, the Results are presented primarily as log_10_ FFU/mL.

### 2.9. Cell Growth Assessment and Culture Monitoring

Cell growth in the macrocarrier-based system was assessed by recovering representative macrocarriers (2–3 macrocarriers per sampling time point), followed by trypsinization and cell counting. Culture status was monitored by measuring glucose concentration and pH in culture supernatants at regular intervals. These parameters were used to assess culture progression, guide infection timing, and define the productive phase of viral replication.

### 2.10. Experimental Design and Endpoints

**Figure 1:**
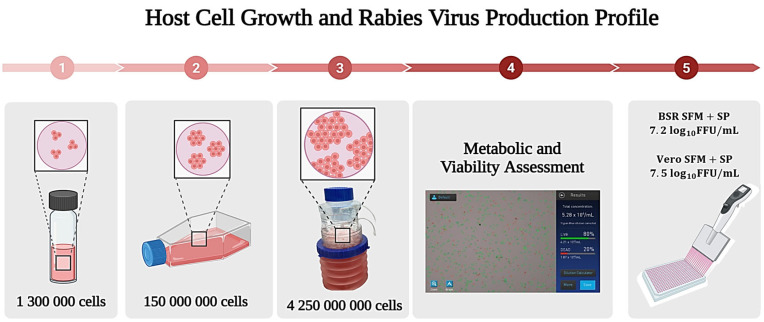
Schematic overview of the experimental workflow in the CelCradle™ macrocarrier-based system and the principal downstream readouts, including cell growth assessment, culture monitoring, and infectious virus titer determination. Numbered steps indicate the experimental workflow: (**1**) expansion of Vero and BSR cells under standard culture conditions; (**2**) harvesting and preparation of single-cell suspensions; (**3**) seeding onto BioNOC™ II macrocarriers; (**4**) high-density cultivation in a tide-motion bioreactor system (CelCradle™); (**5**) infection with rabies virus CVS-11 and subsequent monitoring of virus production and culture parameters.

Experimental conditions included combinations of serum-containing and serum-free media, with or without sodium pyruvate supplementation, evaluated in Vero and BSR cultures cultivated in the macrocarrier-based system and compared with conventional monolayer cultures. Complementary monolayer experiments, including BHK cells, were incorporated to assess post-infection cell-density dynamics across different cell substrates under conventional conditions. The primary endpoints were peak infectious virus titer, duration of the productive phase, and temporal patterns of infectious virus production. Secondary endpoints included cell growth dynamics, post-infection cell-density decline, and culture-monitoring parameters. Figure 1 provides a schematic overview of the experimental framework and illustrates the relationships among high-density cultivation, culture constraints, and virus production outcomes assessed in this study.

### 2.11. Statistical Analysis

Statistical analyses were performed using R software (v4.3.2). Each experimental condition was evaluated in at least three independent biological replicates, defined as separate cultivation runs. For comparisons of peak infectious titers (log_10_ FFU/mL), the effects of serum condition (serum-containing vs. serum-free medium) and sodium pyruvate supplementation were analyzed using two-way factorial ANOVA, including assessment of main effects and their interaction. When appropriate, post hoc multiple-comparison testing was performed using Tukey’s HSD test with adjustment for multiple comparisons. The association between minimum culture pH and peak infectious virus titer was assessed using Pearson’s correlation coefficient. Differences were considered statistically significant at *p* < 0.05 [27,28]. Pairwise comparisons of peak titers between supplemented and non-supplemented conditions were also performed using post hoc multiple comparison tests with adjustment for multiple testing.

## 3. Results

### 3.1. Establishment of High-Density Adherent Cultures in the Tide-Motion Macrocarrier-Based System

Vero and BSR cells successfully attached to BioNOC™ II macrocarriers and formed high-density adherent cultures in the tide-motion macrocarrier-based system. Attachment efficiency exceeded 85% within the first 1–3 h after seeding, enabling subsequent expansion under tide-motion conditions. Following attachment, both cell lines showed sustained proliferation, resulting in dense macrocarrier colonization before virus inoculation.

**Figure 2:**
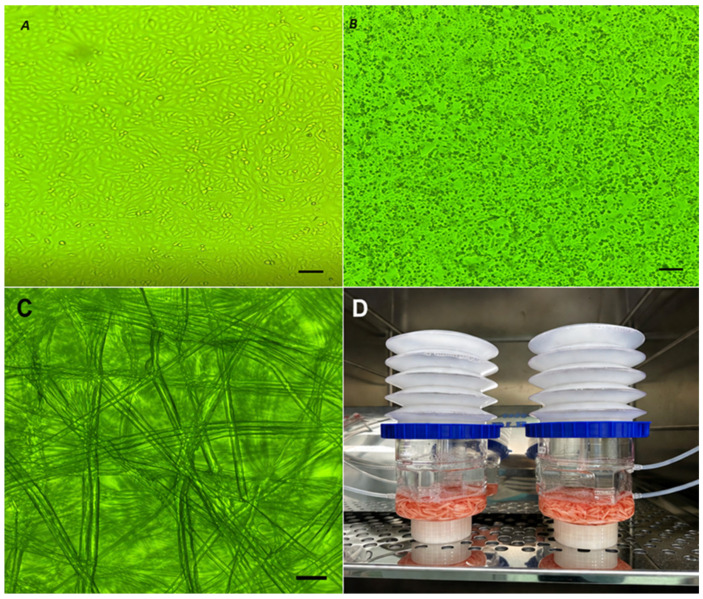
Representative images illustrating Vero cell morphology and rabies virus-induced cytopathic effects under different cultivation conditions. (**A**) Vero cell monolayer at 72 h after seeding under control conditions, showing typical spindle-shaped morphology and uniform distribution. (**B**) Cytopathic effects in Vero cells at 48 h after infection with rabies virus CVS-11, characterized by cell rounding, detachment, and disruption of the monolayer integrity. (**C**) Attachment and growth of Vero cells on BioNOC™ II macrocarriers in the CelCradle™ tide-motion macrocarrier-based system, illustrating high-density adherent growth. (**D**) Photographic view of the CelCradle™ cultivation system used during the experiments. Microscopy images (**A**–**C**) were acquired using an optical phase-contrast microscope. Scale bars: 100 μm.

**Figure 3:**
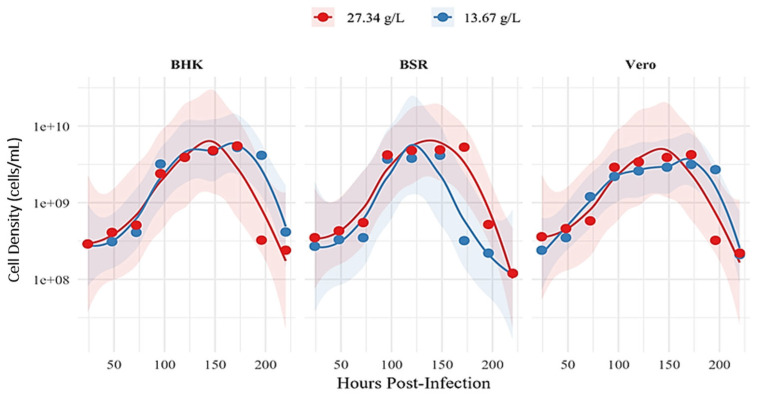
Post-infection cell density dynamics under different cultivation regimens in standard DMEM (13.67 g/L powder per liter) and two-fold concentrated DMEM (27.34 g/L powder per liter), corresponding to standard and double-strength medium formulations. Data points represent mean values obtained from independent biological replicates. Solid lines indicate fitted trends, and shaded regions represent variability around the fitted curves (±standard deviation).

Cell expansion profiles indicated that high cell densities could be achieved and maintained under serum-free conditions, without evident macrocarrier aggregation or extensive cell detachment during cultivation. Representative micrographs and quantitative cell-density data for the high-density macrocarrier-based cultures are shown in Figure 2. To complement these observations, post-infection cell-density dynamics were additionally evaluated in conventional monolayer cultures of Vero, BSR, and BHK cells, as shown in Figure 3.

To further characterize culture behavior during the infection phase, post-infection cell density was quantified under the different cultivation conditions. The temporal profiles are presented in Figure 3.

Cell density was monitored over time after CVS-11 infection and plotted as a function of hours post-infection for each cell line. The fitted curves compare cultures maintained in standard and concentrated DMEM, with shaded regions indicating variability around the fitted trends (see Figure 3 legend for details). The profiles illustrate temporal changes in cell density during the productive phase of infection and the subsequent decline at later time points. These temporal changes in post-infection cell density are consistent with the observed differences in infectious virus yield across cultivation conditions (Table 1).

Across conditions, cultures reached high cell densities before infection and subsequently showed a progressive decline in cell density during the later stage of the productive phase. This pattern was observed in BHK, BSR, and Vero cultures, although the timing and extent of the decline varied among cell lines and cultivation conditions.

### 3.2. Metabolic Profiles Under High-Density Cultivation Conditions

During high-density cultivation, glucose consumption and pH dynamics changed in parallel with cell expansion. In control cultures without sodium pyruvate supplementation, glucose depletion occurred earlier and was accompanied by a progressive decline in pH during the post-infection phase. In contrast, cultures supplemented with sodium pyruvate (1 mM) showed delayed glucose depletion and more stable pH profiles over the cultivation period. Similar trends were observed in both Vero and BSR cultures. Temporal culture-monitoring profiles and the associated production parameters are summarized in Table 1.

Statistical analysis confirmed that sodium pyruvate supplementation was associated with significantly higher peak infectious titers (*p* < 0.05), supporting the observed differences across experimental conditions. To examine how cultivation conditions related to virus productivity, peak infectious titers were compared across serum-containing (SCM) and serum-free (SFM) media, with or without sodium pyruvate supplementation (SP; final concentration 1 mM). Overall, higher peak infectious titers were observed under serum-free conditions and in cultures supplemented with sodium pyruvate. In addition, lower minimum culture pH values coincided with higher peak infectious titers.

Statistical analysis of peak infectious titers confirmed significant effects of both serum condition and sodium pyruvate supplementation on virus productivity. Two-way ANOVA showed a significant main effect of serum condition (F = 58.33, *p* = 0.0015), with higher titers observed under serum-free conditions. Sodium pyruvate supplementation also had a significant main effect (F = 10.71, *p* = 0.0369). No significant interaction between serum condition and pyruvate supplementation was detected (*p* = 0.3365), indicating that the effect of pyruvate was not dependent on the basal medium condition. Representative micrographs and quantitative cell-density data for the high-density macrocarrier-based cultures are shown in Figure 2. To complement these observations, post-infection cell-density dynamics were additionally evaluated in conventional monolayer cultures of Vero, BSR, and BHK cells, as shown in Figure 3.

**Figure 4:**
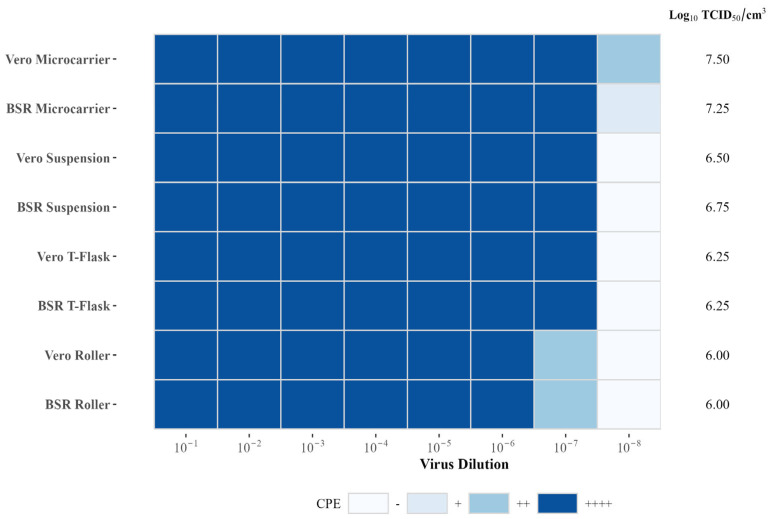
Comparative CPE profiles and infectious activity of rabies virus CVS-11 in Vero and BSR cell cultures under different cultivation conditions. Virus-containing samples from macrocarrier-based and conventional T-flask cultures were subjected to serial 10-fold dilution and evaluated on susceptible cell monolayers. Color intensity indicates the relative extent of detectable cytopathic effect (CPE) across dilution series. The values shown on the right represent the corresponding peak infectious virus titers (log_10_ FFU/mL) for each condition. This figure complements the quantitative infectivity data presented in Table 1 and enables direct comparison of infectivity patterns across cultivation systems.

Figure 4 summarizes the comparative cytopathic effect (CPE) profiles of CVS-11-containing samples from Vero and BSR cultures across serial 10-fold dilutions, together with the corresponding peak infectious titers determined for each condition.

Growth characteristics, culture-monitoring parameters, and rabies virus productivity in Vero and BSR cultures cultivated in the CelCradle™ system under serum-containing (SCM) or serum-free (SFM) conditions, with or without sodium pyruvate supplementation (SP), are summarized in Table 1. Productive days were defined as days during which infectious virus remained detectable, and virus productivity is reported as the maximum infectious titer determined by the FFU assay.

### 3.3. Effect of Sodium Pyruvate on Cell Viability During the Productive Phase

Cell viability during the productive phase of rabies virus infection differed between sodium pyruvate-supplemented and non-supplemented cultures. As shown in Figure 3 and supported by the summarized data in Table 1, in the absence of sodium pyruvate, viable cell numbers declined more rapidly after infection. In contrast, cultures supplemented with sodium pyruvate maintained higher viable cell densities for a longer period during the post-infection phase. This pattern was observed in both cell lines and was most evident during the later stage of the productive phase. These findings are consistent with the prolonged productive phase and higher peak infectious titers observed under pyruvate-supplemented conditions.

### 3.4. Rabies Virus CVS-11 Replication Kinetics in High-Density Macrocarrier-Based System Cultures

Rabies virus CVS-11 replication was observed in both Vero and BSR cells cultivated in the CelCradle™ tide-motion macrocarrier-based system. Across experimental conditions, virus production followed a reproducible kinetic pattern characterized by a productive phase lasting approximately 9–10 days after infection.

Peak viral infectious titer occurred between days 7 and 9 post-infection, depending on the cell line and culture conditions. The time of peak production coincided with a marked decline in viable cell density, consistent with progressive virus-induced cytopathic effects and loss of host-cell integrity. After the peak production period, infectious titers gradually declined in parallel with increasing proportions of non-viable cells.

Post-infection measurements showed substantial reductions in cell numbers in both Vero and BSR cultures, with greater cell loss observed under serum-free conditions. Minimum culture pH values also varied across the infection period and among cultivation conditions. Replication duration, timing of peak infectious virus production, post-infection cell loss, and pH dynamics are summarized in Table 2.

Serum-free cultivation was associated with increased post-infection cell loss (F = 12.00, *p* = 0.0257), whereas sodium pyruvate supplementation significantly reduced cell mortality (F = 8.33, *p* = 0.0447). No significant interaction between serum condition and sodium pyruvate supplementation was detected for post-infection cell mortality (*p* = 0.3125), indicating that the protective effect of pyruvate was consistent across different medium conditions.

Exploratory correlation analysis based on condition-level summary data indicated an inverse association between minimum culture pH and the percentage of dead cells (r = −0.861, *p* = 0.006), suggesting that lower pH values, likely reflecting increased metabolic stress, were associated with reduced cell survival. However, this relationship should be interpreted with caution, as it was derived from aggregated condition-level data rather than replicate-level measurements.

For virus productivity, two-way ANOVA showed a significant main effect of serum condition (F = 58.33, *p* = 0.0015), indicating that serum-free conditions were associated with higher peak infectious titers. Sodium pyruvate supplementation also had a significant main effect (F = 10.71, *p* = 0.0369), supporting its role in enhancing virus production. No significant interaction between serum condition and sodium pyruvate supplementation was detected (*p* = 0.3365), suggesting that the effect of pyruvate on virus yield was consistent across different medium conditions.

Replication characteristics of rabies virus CVS-11 in Vero and BSR cultures cultivated in the CelCradle™ system under serum-containing (SCM) and serum-free (SFM) conditions, with or without sodium pyruvate supplementation (SP; final concentration 1 mM), are summarized in Table 2. The measured parameters included replication duration, timing of peak infectious virus production, post-infection cell loss, and culture pH dynamics.

Across conditions, serum-free cultivation was associated with increased post-infection cell loss. Lower minimum culture pH values also coincided with higher proportions of dead cells.

### 3.5. Comparison with Conventional Monolayer Cultivation Systems

Parallel control experiments in conventional T-flask monolayer cultures showed lower maximum infectious titers and a shorter productive phase than those observed in high-density macrocarrier-based cultures. Under serum-free conditions, monolayer cultures also showed an earlier decline in culture conditions and a more rapid loss of viable cells after infection.

When compared directly, high-density macrocarrier-based cultivation provided higher volumetric virus productivity and a longer virus production window than conventional monolayer systems. In addition to differences in virus output, the cultivation formats also differed in their growth context, including achievable cell density, working volume, and serum requirement. A comparative summary of key biological and operational characteristics of selected cultivation systems relevant to rabies virus propagation is presented in Table 3.

**Figure 5:**
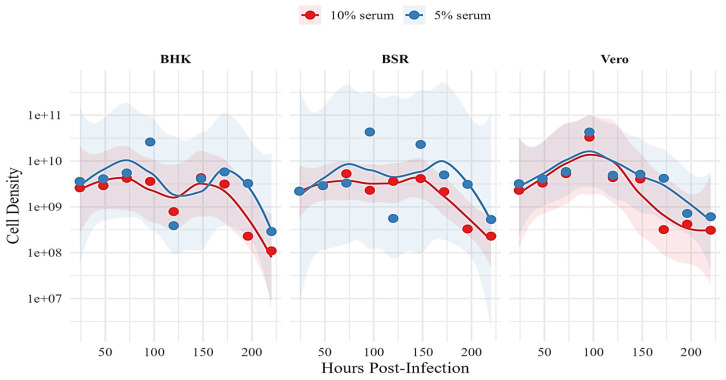
Post-infection cell density dynamics in monolayer cultures maintained under different serum concentrations. Data points represent mean values from independent biological replicates. Solid lines indicate fitted trends, and shaded regions represent ± standard deviation (SD) around the fitted curves. Red and blue colors correspond to cultures maintained in 10% and 5% serum, respectively. The x-axis represents hours post-infection, and the y-axis represents cell density (cells/mL).

To complement the comparative overview in Table 3, post-infection cell density dynamics were evaluated in monolayer cultures maintained under different serum concentrations. The temporal profiles are shown in Figure 5. BHK monolayer cultures were included as an additional reference cell substrate in these serum-comparison profiles.

Cell density was monitored over time after CVS-11 infection and plotted as a function of hours post-infection for each cell line. The fitted curves compare cultures maintained in 10% and 5% serum, with shaded regions indicating variability around the fitted trends. The profiles illustrate temporal changes in cell density during the productive phase of infection and the subsequent decline at later time points.

Across cell lines, lower serum concentration was associated with earlier and/or more pronounced decline in cell density during the post-infection period. This pattern was observed in BHK, BSR, and Vero monolayer cultures, although the magnitude of the difference varied among cell lines.

## 4. Discussion

Although host-cell metabolism is widely recognized as an important determinant of virus production, its role under high-density adherent culture conditions remains insufficiently characterized, particularly in systems where nutrient availability, waste accumulation, and density-associated cellular stress differ substantially from those observed in conventional monolayer cultures [29,30,31]. Most previous studies on rabies virus cultivation have primarily emphasized process optimization and vaccine production efficiency. In contrast, less attention has been given to how culture-associated physiological constraints influence the ability of infected cells to sustain infectious virus output over time [32,33,34]. In the present study, we addressed this question using a tide-motion macrocarrier-based culture system as a controlled high-density environment and evaluated whether sodium pyruvate supplementation could improve infectious CVS-11 yield by supporting host-cell condition during the post-infection phase.

Across the tested conditions, sodium pyruvate supplementation was associated with delayed culture deterioration, prolonged maintenance of viable cell populations, and higher peak infectious titers in both Vero and BSR cultures. These effects were observed under both serum-containing and serum-free conditions, with the highest infectious titers recorded in serum-free pyruvate-supplemented cultures. Importantly, because all cultures were infected at the same MOI and evaluated within the same general experimental framework, the differences observed are more consistently interpreted as reflecting changes in host-cell performance during the productive phase rather than variation in virus input. Under this interpretation, sodium pyruvate supplementation appears to support a culture state that remains compatible with sustained infectious virus production for longer after infection.

However, the observed effects of sodium pyruvate should be interpreted with appropriate caution. Sodium pyruvate is a central intermediate of cellular carbon metabolism and may contribute to the maintenance of cellular bioenergetic balance under metabolically demanding conditions [35]. In the present study, pyruvate-supplemented cultures showed delayed glucose depletion, altered pH trajectories, improved maintenance of viable cell populations, and an extended productive phase, consistent with the interpretation that pyruvate helped stabilize host-cell condition during infection [36,37]. At the same time, the current dataset does not provide direct mechanistic evidence that pyruvate specifically altered the intracellular replication machinery of CVS-11. No direct measurements of intracellular metabolites, mitochondrial activity, redox balance, lactate accumulation, or related metabolic endpoints were performed. Therefore, the present findings should be viewed as evidence of improved infectious virus production associated with metabolic support, rather than as direct proof of a defined metabolic mechanism controlling rabies virus replication.

Although CVS-11 is primarily used as a challenge strain, it has also been applied in certain vaccine-related contexts. The observed effects of sodium pyruvate supplementation on host-cell viability and infectious virus yield are likely to reflect general metabolic mechanisms relevant to rabies virus replication in vitro. However, the extent to which these findings can be directly extrapolated to other rabies virus strains may depend on strain-specific biological properties and should be interpreted with appropriate caution.

An additional strength of the study is the inclusion of multiple cell substrates within the overall experimental design. Vero and BSR cells served as the principal host systems for high-density macrocarrier-based cultivation and virus-production analysis. In contrast, BHK cells were included in comparative monolayer experiments to contextualize post-infection cell-density responses across different cell substrates. Although the magnitude and timing of post-infection decline differed among cell lines and cultivation settings, the overall pattern was consistent. Once infection progressed, deterioration of host-cell condition coincided with declining cell density and subsequent reduction in infectious virus output. This reinforces the conclusion that cultural context and maintenance of viable producer-cell populations are closely linked to the duration of productive infection.

The comparison with conventional monolayer cultivation further supports this interpretation. Monolayer cultures showed lower maximum infectious titers and a shorter productive phase than the high-density macrocarrier-based system, particularly under serum-free conditions. These findings suggest that the macrocarrier-based tide-motion system offers not only technological advantages in terms of volumetric output but also a more favorable environment for sustaining infected adherent cells during the virus-production phase. In this respect, the system should be viewed not solely as a scale-up platform, but also as an experimentally informative model for examining how dense-culture conditions shape infectious virus production in rabies virus-permissive cell substrates.

The correlation analyses involving minimum culture pH should also be interpreted conservatively. Although inverse associations were observed between minimum pH and both peak infectious titer and post-infection cell mortality, these analyses were based on eight aggregated condition-level observations rather than replicate-level measurements. Accordingly, they should be regarded as exploratory and supportive, rather than as independent evidence of a causal relationship between culture acidification and virus-production outcomes.

Several limitations should be acknowledged. First, the study focused on sodium pyruvate as a single defined metabolic intervention, whereas infectious rabies virus production is likely influenced by a broader set of metabolic and physicochemical variables. Second, the absence of direct intracellular metabolic measurements limits mechanistic resolution. Third, although BHK cells were included to strengthen the comparative interpretation of post-infection cell-density behavior, virus-production analyses in the high-density macrocarrier-based system were centered on Vero and BSR cells. Future studies integrating targeted metabolic profiling, intracellular virological readouts, and more detailed time-resolved analyses would help clarify how host-cell support strategies can be used to stabilize and improve CVS-11 production in adherent culture systems.

## 5. Conclusions

Under the experimental conditions used in this study, sodium pyruvate supplementation was associated with prolonged maintenance of viable cell populations, an extended productive phase, and higher rabies virus CVS-11 infectious titers in high-density macrocarrier-based cultures. These findings support sodium pyruvate supplementation as a practical culture-modulation strategy for improving infectious rabies virus yield in adherent cell systems and provide a basis for further studies integrating virological and metabolic analyses in high-density production settings.

## Figures and Tables

**Table 1:** Effects of culture conditions, including serum availability and sodium pyruvate supplementation, on cell growth, culture-monitoring parameters, and rabies virus production in the CelCradle™ bioreactor. Glucose values are presented as observed ranges during cultivation and infection, reflecting changes in cellular metabolism and nutrient consumption. Abbreviations: SCM, serum-containing medium; SFM, serum-free medium; SP, sodium pyruvate.

Parameter	Vero SCM	Vero SCM + SP	Vero SFM	Vero SFM + SP	BSR SCM	BSR SCM + SP	BSR SFM	BSR SFM + SP
Culture duration (days)	5	4	4	4	5	5	5	4
BioNOC II™ (g)	5.5	5.5	5.5	5.5	5.5	5.5	5.5	5.5
Day of peak cell density	7	7	9	8	8	7	7	7
Max. total cells (×10^9^)	2.1	2.4	4.2	4.5	2.3	2.8	3.9	4.3
Cells/mL (×10^6^)	2.5	2.2	3.5	5.2	2.2	2.3	3.3	5.0
Cells per macrocarrier (×10^6^ cells/carrier)	2.2	2.4	5.0	5.1	2.7	2.4	2.9	3.5
Glucose (mg/dL)	200–400	200–350	180–250	160–340	250–400	200–350	280–400	160–320
pH range	7.0–7.2	6.5–6.9	6.1–7.1	5.9–6.9	6.5–7.1	6.6–6.8	6.2–7.1	5.7–6.1
Harvest volume (L)	2.5	2.5	2.5	2.5	2.5	2.5	2.5	2.5
Productive days	10–11	11–12	11–12	11–12	10–11	10–11	10–11	11–12
Max. virus titer (log_10_ FFU/mL)	6.0	6.7	7.2	7.5	6.2	6.5	7.0	7.2

Note: “Culture duration” refers to the duration of the defined cultivation phase under the specified conditions, whereas “Day of peak cell density” indicates the time point at which the highest cell density was observed during the overall monitoring period; reported time points correspond to the full cultivation timeline, including both pre- and post-infection phases.

**Table 2:** Replication characteristics of rabies virus CVS-11 in the CelCradle™ macrocarrier-based system.

Parameter	Vero SCM	Vero SCM + SP	Vero SFM	Vero SFM + SP	BSR SCM	BSR SCM + SP	BSR SFM	BSR SFM + SP
CVS-11 replication period (days)	9–10	9–10	9–10	9–10	9–10	9–10	9–10	9–10
Medium exchange (every 24 h)	1	1	1	1	1	1	1	1
BioNOC II™ macrocarriers (g)	5.5	5.5	5.5	5.5	5.5	5.5	5.5	5.5
Day of peak virus production	7	7	9	8	8	7	7	7
Cell density decline after infection (cells/mL, BelloCell 500-AP)	1.5 × 10^6^	1.8 × 10^6^	1.2 × 10^6^	1.5 × 10^6^	1.3 × 10^6^	1.4 × 10^6^	1.8 × 10^6^	2.3 × 10^6^
Cells/mL (post-infection)	1.5 × 10^5^	1.3 × 10^5^	1.0 × 10^5^	0.9 × 10^5^	1.2 × 10^5^	0.9 × 10^5^	1.2 × 10^5^	0.8 × 10^5^
Cells per macrocarrier (post-infection)	1.5 × 10^5^	1.2 × 10^5^	0.8 × 10^5^	1.1 × 10^5^	1.1 × 10^5^	1.0 × 10^5^	0.9 × 10^5^	1.0 × 10^5^
Dead cells after CVS-11 infection (%)	72	81	84	87	78	83	82	85
pH range during infection	7.0–7.2	6.5–6.9	6.1–7.1	5.9–6.9	6.5–7.1	6.6–6.8	6.2–7.1	5.7–6.1

**Table 3:** Comparative biological and operational characteristics of selected cell cultivation systems relevant to rabies virus propagation.

Parameter	Macrocarrier-Based System (CelCradle™)	Suspension Culture	T-flask Monolayer	Roller Bottle
Achievable cell density (cells/mL)	5.3 × 10^6^	3.2 × 10^6^	2.9 × 10^6^	3.7 × 10^6^
Total culture medium volume per run (L)	2.0	20.0	12.5	6.0
Serum requirement (mL)	200	2000	1200	600
Cultivation duration (h)	192	168	96	120
Growth format	High-density adherent	Suspension	Adherent monolayer	Adherent monolayer
Surface-to-volume ratio	High	Low	Moderate	Moderate

## Data Availability

The data presented in this study are available in the article.

## Abbreviations

The following abbreviations are used in this manuscript:

CPE	cytopathic effect(s);
DMEM	Dulbecco’s modified Eagle medium;
FFU	focus-forming units;
MOI	multiplicity of infection;
SCM	serum-containing medium;
SP	sodium pyruvate;
TCID_50_	50% tissue culture infectious dose.
